# Microbial Primer: Microbiome and thermal tolerance – a new frontier in climate resilience?

**DOI:** 10.1099/mic.0.001523

**Published:** 2025-01-21

**Authors:** Jingdi Li, Kayla King

**Affiliations:** 1 Department of Zoology, University of British Columbia, Vancouver, Canada; 2 Department of Biology, University of Oxford, Oxford, UK

**Keywords:** climate change, conservation, microbiome, symbiosis, temperature, thermal tolerance

## Abstract

Microbiome-animal host symbioses are ubiquitous in nature. Animal-associated microbiomes can play a crucial role in host physiology, health and resilience to environmental stressors. As climate change drives rising global temperatures and increases the frequency of thermal extremes, microbiomes are emerging as a new frontier in buffering vulnerable animals against temperature fluctuations. In this primer, we briefly introduce key concepts of microbiome-host symbiosis and microbial responses to temperature shifts. We then summarize the current evidence and understanding of how microbes can buffer the thermal stress faced by their hosts. We identify key challenges for future research. Finally, we emphasize the potential of harnessing microbiomes to improve conservation strategies in a rapidly changing climate, offering a concise overview of this evolving field.

## Microbiome-host symbiosis

In nature, bacteria can have a free-living lifestyle or form associations with hosts across the tree of life. Microbe-host associations are ubiquitous across the tree of life, and many have evolved to benefit both partners. These mutualistic interactions have been extensively studied in insect hosts and their endosymbionts, such as pea aphid (*Acyrthosiphon pisum*) and its obligate bacterial endosymbiont *Buchnera aphidicola*.

In recent years, advances in sequencing technology have uncovered a far more complex and diverse microbial community associated with host organisms. Virtually all animals and plants host an abundance of microorganisms – mostly bacterial, together with archaeal, fungal and viral communities residing inside and on host surfaces. Of these, the bacterial component – often simply referred to as the ‘microbiome’ – has been the focus of intensive research. In the last decade, a vast array of previously uncultured bacterial species has been revealed. Host microbiomes can profoundly influence host health through modulating physiology, behaviour, nutrition and immune function. Animals that develop without microbiomes can exhibit delayed development, stunted growth, reduced fecundity or shortened lifespan. More recently, microbiome research has further expanded to explore the role of microbiomes in protecting hosts from environmental stressors, such as high temperatures.

## Microbial response to temperature

Temperature plays a fundamental role in the cellular processes of bacteria, including the behaviour of DNA, RNA, protein and lipids. Bacteria rely on external temperatures to regulate their metabolic processes. Within their normal temperature range, bacterial growth rates generally follow a linear relationship with temperature, which can be described by the Arrhenius equation. Bacterial growth rates peak at an optimal temperature, which varies between species. For *Escherichia coli*, the ‘Arrhenius’ range spans from 23 to 37 °C, with 37 °C as the optimum. Within this range, *E. coli* grows efficiently, and temperature shifts only induce minor adjustments to protein production. However, temperatures outside this range cause stress, leading to protein misfolding and disrupted nucleic acid (DNA and RNA) conformation, causing minimal or ceased bacterial growth.

Microbes employ various mechanisms to cope with environmental stresses, such as thermal stress, including both general stress response and temperature-specific responses. The general stress response is a broad-spectrum strategy that enables bacteria to withstand multiple stressors, including heat and cold. This response is often regulated by alternative sigma factors, such as RNA polymerase sigma factor in Gram-negative bacteria, which activates the expression of genes involved in DNA repair and the production of antioxidant enzymes. In contrast, temperature-specific responses involve the induction of heat shock and cold shock proteins. The former can function as molecular chaperones, assisting in repairing the misfolded proteins, as well as proteases, which degrade irreparably damaged proteins.

## Animal thermal tolerance and microbial changes

**Fig. 1.**
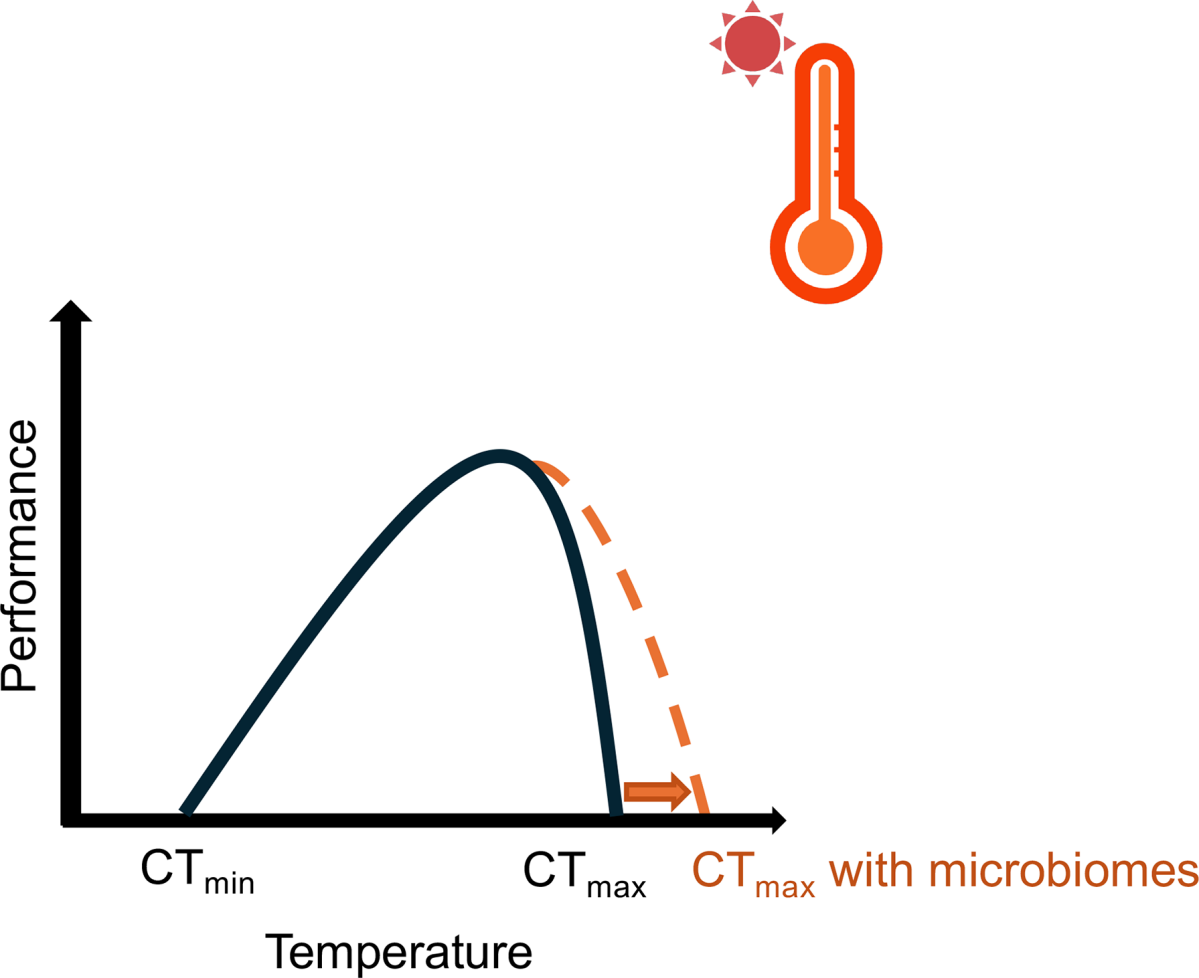
The microbiome has the potential to extend host thermal tolerance, a phenomenon extensively documented in ectothermic animals. The central panel depicts the animal thermal tolerance curve, characterized by lower (CT_min_) and upper thermal limits (CT_max_). Host-associated microbiomes can buffer the host against warming stress, shifting CT_max_ rightward. This shift expands the range of thermal tolerance and enhances host resilience to temperature extremes. Created with BioRender.

As climate change accelerates global warming and increases the frequency of extreme thermal events, many animal species face heightened risks of extinction. Thermal tolerance, characterized by upper and lower thermal limits (Fig. 1), is a critical factor influencing species ecology and evolution. One key parameter used to assess upper thermal tolerance is the critical thermal maximum (CT_max_), first defined by Cowles and Bogert (1944) as the temperature at which an animal’s locomotion becomes disorganized and loses its ability to escape from lethal conditions during thermal ramping. CT_max_ was initially developed for ectotherms. For these animals, body temperatures fluctuate with environmental conditions, making them especially vulnerable to warming. Ectotherms rely heavily on suitable microclimates and behavioural strategies, such as seeking shade, to regulate their body temperatures. Alternatively, endotherms generate heat internally through metabolic processes. This ability allows them to maintain a relatively stable body temperature independent of external temperatures. Prolonged thermal stress leads to increased energy expenditure, ultimately reducing fitness or causing death in endothermic animals. Thermal tolerance provides valuable insight into how animals may respond to climate change, particularly for species currently living near their thermal maximum.

Traditionally, studies of thermal tolerance have focused on animals’ behavioural and physiological responses, including their plasticity in coping with thermal stress (see Johnston and Bennett 2008 in the reading list for further information). The heat shock response, involving the upregulation of heat shock genes and activation of the heat shock transcription factor 1, is conserved across ectothermic and endothermic animals. Endotherms possess more complex regulatory systems for thermal control, involving hormone pathways and brown adipose tissue thermogenesis.

Emerging evidence points to the critical role of microbiomes in modulating animal thermal tolerance, especially for ectotherms (Fig. 1). For example, depletion of gut microbiome diversity in tadpoles reduced their thermal tolerance, leading to decreased survival under acute heat stress. Since ectotherms’ body temperatures fluctuate with environmental conditions, their microbiomes are directly exposed to the ambient temperature changes. These microbial communities, characterized by large population sizes and rapid metabolic rates, can quickly respond to temperature fluctuations through shifts in species composition and abundance.

Microbiome responses to temperature are often evaluated through community-level traits, such as diversity and composition. Two key metrics commonly used to assess microbial community changes are alpha and beta diversity. Alpha diversity measures species richness and evenness within a single community, providing insight into the variety of bacterial species. Beta diversity compares differences in microbiome composition between communities or samples. Elevated temperatures can reduce microbial diversity across a wide range of host species. A loss of diversity can be linked to negative health outcomes for the host, such as reduced digestive capacity in salamanders or lower survival rates in lizards. Shifts in microbiome diversity are common in organisms experiencing physiological stress, such as marine species facing warming or humans with poor health. In addition to diversity shifts, compositional changes – including increase and decrease in the abundance of specific taxa – are frequently observed under thermal stress. For example, warming has been shown to increase microbiome variability in sponges and *Caenorhabditis elegans*, while also promoting the abundance of pathogenic bacteria within coral microbiomes.

These community-level shifts in microbiomes have been used as indicators of altered host health and fitness, highlighting them as important components in host acclimation and adaptation to environmental stress. Microbial changes can also enhance a host’s ability to withstand temperature extremes, a phenomenon well-documented in coral systems. Compositional shifts in coral-associated microbial communities have been shown to buffer corals against warming, and the application of probiotics – beneficial microbes that support host health – has been identified as a potential strategy to protect corals from heat stress-induced bleaching.

## Increased host thermal tolerance mediated by microbiomes

Symbionts can mediate host thermal tolerance via different mechanisms. They can upregulate the expression of host stress-response genes or produce protective metabolites within hosts. For example, *Rickettsia* protects the whitefly (*Bemisia tabaci*) from heat by inducing stress-response gene expression at moderate temperatures, which primes the host to better cope when warming temperatures occur. In corals, probiotic bacterial species have been shown to mitigate the negative effect of high temperatures by facilitating dimethyl sulfoniopropionate degradation which maintains lipid homeostasis. These coral symbionts can also reprogram host transcription of cellular restructuration, repair, stress protection and immune genes during warming.

The role of complex microbiomes – composed of diverse species with varying functions – in mediating thermal tolerance is less resolved. The microbiome-mediated thermal tolerance in tadpoles was found to be linked to the upregulation of genes involved in amino acid biosynthesis and catabolism, with the underlying mechanisms unclear. Microbiome transplants have provided valuable insight into the degree to which microbiomes contribute to thermal tolerance in ectothermic and endothermic hosts. Microbiome transplants from heat-tolerant *Drosophila melanogaster* improved the capacity of recipient flies to better tolerate elevated temperatures. Transplants from heat- or cold-exposed hosts can modulate energy dissipation and thermogenesis ultimately increasing the mouse’s ability to better cope with heat or cold, respectively. Specifically, mice receiving microbiomes from heat-exposed donors exhibited an increase in large unilocular adipocytes, while those receiving microbiomes from cold-exposed donors showed a higher number of small adipocytes.

## Microbiome and climate change: a new conservation tool?

Understanding the role of microbiomes in mediating thermal tolerance offers an exciting potential for conservation strategies in the face of climate change. Microbiomes can serve as an early detection tool, allowing forecasts of host health before adverse symptoms appear. This approach has been developed in coral ecosystems, where shifts in coral microbiomes are predictive of health declines under ocean warming.

Going forward, as global temperatures continue to rise and habitats degrade, microbiome-based interventions may be a viable strategy to help protect vulnerable species. Microbiome transplants from heat-tolerant individuals to more sensitive hosts have shown promise in enhancing thermal resilience in a variety of species, ranging from fruit flies to mice. Additionally, screening for protective microbial species to develop targeted probiotics could mitigate thermal stress in increasingly threatened or endangered animals.

## Gaps in knowledge and future research directions

Despite the great potential of microbiomes to support animal conservation under climate warming, significant gaps in our understanding remain. First, most (if not all) evidence supporting the role of microbiomes in animal thermal tolerance comes from laboratory experiments and is limited to a narrow range of host species, primarily invertebrate ectotherms. Invertebrates are easier to manipulate in the lab which may explain their widespread use as model organisms in host-microbe interaction studies. To generalize these findings across diverse animal taxa, it is crucial to expand research to include a broader range of species. Testing whether microbiomes might facilitate thermal tolerance in endothermic vertebrates might hint at ways endangered mammals (e.g. polar bears) and even humans may better cope with warming climates. Beyond the lab, more field-based studies are needed to assess how microbiomes and host thermal responses function in the more complex and dynamic conditions of natural environments. For example, invertebrates with complex life cycles could experience temperature variability at different life stages in the wild. Understanding how microbiome establishment and function buffer these species in these thermal conditions is essential for applying microbiome research to real-world conservation efforts.

Second, the mechanisms through which shifts in microbial diversity and composition affect host thermal tolerance remain poorly understood. In particular, microbiome transplant studies have yet to clarify whether enhanced thermal tolerance is driven by overall diversity, specific microbial taxa or associated functional pathways. Without a clear understanding of the mechanisms at play, the long-term efficacy of microbiome transplants for improving host thermal tolerance may not be ensured. Microbial populations can evolve rapidly within a host’s lifespan, potentially altering their protective traits (e.g. compositions and functions). Future studies should prioritize longer-term experiments that track both transplanted microbiomes and host responses to fluctuating temperatures.

Finally, there may be trade-offs between microbiome-mediated thermal tolerance and key host life-history traits (such as fecundity). Whether the thermal protection provided by microbes comes at a cost to host fecundity or development may significantly impact their practical use in conservation. Investigating these potential trade-offs is essential to fully understand the benefits and limitations of microbiome interventions.

## Conclusion

The exploration of microbiomes in relation to host thermal tolerance represents a rapidly advancing frontier, with the potential to reshape our understanding of how organisms can acclimate and adapt to climate change. As this field continues to grow, the major challenge will be translating scientific discoveries into practical conservation strategies that promote the long-term health and stability of ecosystems. This effort will require careful consideration of ecological complexity, species-specific responses and long-term sustainability.

## Further reading

1. Hector, T. E., Hoang, K. L., Li, J., & King, K. C. (2022). Symbiosis and host responses to heating. Trends in Ecology & Evolution, 37(7), 611–624. https://doi.org/10.1016/j.tree.2022.03.011
2. Johnston, I. A., and A. F. Bennett, eds. 2008. Animals and temperature. Cambridge, UK: Cambridge Univ. Press
3. Obeng, N., Bansept, F., Sieber, M., Traulsen, A., & Schulenburg, H. (2021). Evolution of Microbiota–Host Associations: The Microbe’s Perspective. Trends in Microbiology, 29(9), 779–787. https://doi.org/10.1016/j.tim.2021.02.005
4. Li, J., Bates, K. A., Hoang, K. L., Hector, T. E., Knowles, S. C. L., & King, K. C. (2023). Experimental temperatures shape host microbiome diversity and composition. Global Change Biology, 29(1), 41–56. https://doi.org/10.1111/gcb.16429
5. Santoro, E. P., Borges, R. M., Espinoza, J. L., Freire, M., Messias, C. S. M. A., Villela, H. D. M., Pereira, L. M., Vilela, C. L. S., Rosado, J. G., Cardoso, P. M., Rosado, P. M., Assis, J. M., Duarte, G. A. S., Perna, G., Rosado, A. S., Macrae, A., Dupont, C. L., Nelson, K. E., Sweet, M. J., … Peixoto, R. S. (2021). Coral microbiome manipulation elicits metabolic and genetic restructuring to mitigate heat stress and evade mortality. Science Advances, 7(33), eabg3088. https://doi.org/10.1126/sciadv.abg3088
6. Fontaine, S. S., & Kohl, K. D. (2023). The microbiome buffers tadpole hosts from heat stress: A hologenomic approach to understand host–microbe interactions under warming. *J. Exp. Biol., 226*, jeb245191. https://doi.org/10.1242/jeb.245191
7. Baldassarre, Laura, et al. "Microbiota mediated plasticity promotes thermal adaptation in the sea anemone Nematostella vectensis." Nature communications 13.1 (2022): 3804.
8. Moghadam, N. N., et al. (2018). Strong responses of *Drosophila melanogaster* microbiota to developmental temperature. *Fly, 12*, 1–12.
9. Bestion, E., Jacob, S., Zinger, L., Di Gesu, L., Richard, M., White, J. and Cote, J. (2017). Climate warming reduces gut microbiota diversity in a vertebrate ectotherm. Nat. Ecol. Evol. 1, 161. https://doi.org/10.1038/s41559-017-0161
10. West, A. G., Waite, D. W., Deines, P., Bourne, D. G., Digby, A., McKenzie, V. J., & Taylor, M. W. (2019). The microbiome in threatened species conservation. Biological Conservation, 229, 85–98. https://doi.org/10.1016/j.biocon.2018.11.016

